# Mouse Models of Aneuploidy

**DOI:** 10.1100/2012/214078

**Published:** 2012-01-03

**Authors:** Olivia Sheppard, Frances K. Wiseman, Aarti Ruparelia, Victor L. J. Tybulewicz, Elizabeth M. C. Fisher

**Affiliations:** ^1^Department of Neurodegenerative Disease, UCL Institute of Neurology, Queen Square, London WC1N 3BG, UK; ^2^Division of Immune Cell Biology, MRC National Institute for Medical Research, The Ridgeway, Mill Hill, London NW7 1AA, UK

## Abstract

Abnormalities of chromosome copy number are called aneuploidies and make up a large health load on the human population. Many aneuploidies are lethal because the resulting abnormal gene dosage is highly deleterious. Nevertheless, some whole chromosome aneuploidies can lead to live births. Alterations in the copy number of sections of chromosomes, which are also known as segmental aneuploidies, are also associated with deleterious effects. Here we examine how aneuploidy of whole chromosomes and segmental aneuploidy of chromosomal regions are modeled in the mouse. These models provide a whole animal system in which we aim to investigate the complex phenotype-genotype interactions that arise from alteration in the copy number of genes. Although our understanding of this subject is still in its infancy, already research in mouse models is highlighting possible therapies that might help alleviate the cognitive effects associated with changes in gene number. Thus, creating and studying mouse models of aneuploidy and copy number variation is important for understanding what it is to be human, in both the normal and genomically altered states.

## 1. Introduction

Traditionally, aneuploidy was defined as a deletion or duplication of a whole chromosome. This genomic abnormality is thought to occur in at least 5% of all clinically recognized pregnancies, usually resulting in spontaneous abortion [1]. Aneuploidy is thought to be usually highly deleterious because many genes are “dosage-sensitive” in that their expression is affected by their copy number in the genome, and changes in gene expression levels may result in altered phenotypes that can be lethal [2]. As well as whole chromosome aneuploidy, deletion of a few kilobases or megabases of  DNA (microdeletion) or similarly a duplicated region (microduplication) within a chromosome can also result in changes in gene copy number. Recent advances in genomic technologies have revealed the association of many of these segmental aneuploidies (microdeletions and duplications) with specific genetic syndromes and diseases [3–5].

The most frequently occurring full autosomal aneuploidy is that of trisomy of human chromosome 21 (Hsa21), which causes Down syndrome (DS). DS is the most common cause of genetic intellectual disability, occurring in ~1 in 750 live births in all populations. People with DS have an increased risk of developing cardiac defects, certain leukemias, and early onset Alzheimer's disease as well as many other phenotypes [6]. Trisomies of chromosomes 18 (Hsa18) (Edwards syndrome) and 13 (Hsa13) (Patau syndrome) occur at lower frequency than DS (1 in 4300 and 1 in 7100 live births, respectively), and infants with these conditions have a very short life expectancy, typically less than 1 year and less than 5 years, respectively [7]. Aneuploidy of the sex chromosomes can occur with multiple copies of the X or Y chromosome, or loss of the X or Y chromosome. Relatively common sex chromosome aneuploidies include Klinefelter syndrome (KS) (47 XXY, males with an additional copy of the X chromosome), ~1 in 500–1000 males [8], and Turner syndrome (TS) (45,X, females with monosomy of the X chromosome), 1 in ~4000 live births [9].

Segmental aneuploidies, otherwise known as partial aneuploidies or segmental aneusomies may be more compatible with life than whole chromosomal aneuploidies, and result in a large number of well-defined syndromes (Table 1). Many of these conditions are associated with neurodevelopmental and growth problems that result in epilepsy, intellectual disability, and autism.

The challenge facing scientists and clinicians from the aneuploidy syndromes is how to unravel the interaction between abnormal gene dosage and abnormal gene expression that leads to the specific phenotypes of each syndrome, and then to find therapies for intervention for these phenotypes.

## 2. Mouse Models of Aneuploidy

The use of mouse models of aneuploidy allows scientists to study the direct effects of abnormal gene dosage on specific syndromes, at the molecular, cellular, physiological, and behavioural level. Many technologies exist to manipulate the mouse genome to mutate, overexpress, and knock-out specific genes of interest, and help to define which dosage sensitive genes are causative for any given phenotype (reviewed in [10]). Such technologies now include chromosome engineering whereby large regions of the mouse genome can be deleted or duplicated corresponding to the partial aneuploidies found in humans (reviewed in [11, 12]). However, one confounding factor is that each human chromosome has syntenic regions to two or more mouse chromosomes. An alternative approach has been to transfer an entire human chromosome into a mouse, to overcome this problem [13]. Here, we discuss the contribution of mouse models of whole chromosome and segmental aneuploidy to our biological understanding and highlight possible future models and how they may further our knowledge.

## 3. Mouse Models of Whole Chromosome Aneuploidies

### 3.1. Down Syndrome

**Figure 1:**
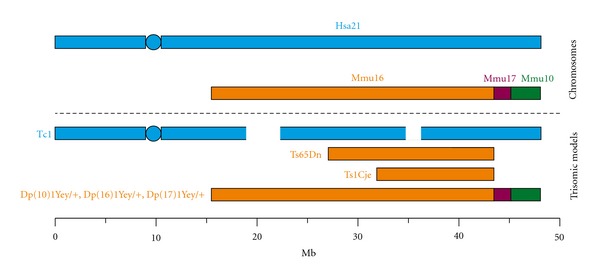
Mouse models of Down syndrome. Hsa21 (in blue) and the syntenic mouse chromosomes (Mmu 16, orange, Mmu 17, purple, Mmu10, green). The trisomic regions of several of the well-established mouse models of DS, the Tc1 mouse, Ts65Dn, Ts1Cje, and Dp(10)1Yey/+, Dp(16)1Yey/+, Dp(17)1Yey/+ are aligned to the corresponding parts of the human and mouse genome.

A number of mouse models have been developed to study the most frequently occurring autosomal aneuploidy, Down syndrome (Figure 1). The Tc1 transchromosomic mouse model contains a freely segregating maternally inherited copy of Hsa21 and is trisomic for approximately 75% of Hsa21 genes [13]. This mouse has altered learning and memory, synaptic plasticity, a reduced cerebellar neuronal number, heart anomalies, reduced solid tumor development, and defects in angiogenesis and megakaryopoiesis [13–18]. Other mouse models of DS contain an additional copy of regions of mouse chromosomes 16, 17, and 10, which are syntenic with Hsa21. The Ts65Dn mouse model is the most widely used; it contains an extra copy of a segment of mouse chromosome 16 (Mmu16) and is trisomic for about 50% of the genes found on Hsa21 [19]. This model shows impaired learning and motor deficits [19], neuronal degeneration similar to that observed in people with Alzheimer's disease (which is part of the DS phenotype) and heart and angiogenesis defects [20–22]. Another commonly used model is the Ts1Cje mouse which contains a smaller segmental trisomy of Mmu16 including approximately 68 genes; it also exhibits learning and behavioral deficits, but does not exhibit neuronal degeneration [23]. The newest model of DS, developed by Yu and colleagues, contains three copies of all Hsa21 homologs on mouse chromosomes 16, 17, and 10 and shows learning and memory deficits that may be similar to some of the cognitive problems that people with DS experience [24, 25].

To determine the identity of trisomic genes that cause specific phenotypes, aneuploid mouse models of DS can be crossed with mouse models of segmental Hsa21 monosomy (Ms1Yah and Ms4Yah) [26–29] or to gene knockouts to alter dosage of individual genes within a region of trisomy. These techniques have been recently used to identify the genes responsible for trisomy-21-related protection against the tumour formation [30], furthering our understanding of the biology that underlies these important processes. Mouse models of Hsa21 trisomy have been used also for demonstrating the potential for cognitive enhancement therapies for people who have DS [21, 31, 32]. A number of the drugs tested in studies of DS mouse models for their effects on learning and memory are currently in small-scale clinical trials, demonstrating the utility of these mice to combat the deleterious effects of DS.

### 3.2. Edwards Syndrome and Patau Syndrome

A mouse model of Edwards syndrome has yet to be developed. Hsa18 is 78 Mb in length and has conserved synteny with 5 principal regions encoded on three mouse chromosome (Mmu 1, 17, and 18). Similarly, no animal model of Patau syndrome has been reported; Hsa13 has conserved synteny with six mouse chromosome segments. Thus, although technically challenging, it would be possible to generate models of these syndromes by duplication of the mouse syntenic regions. These models could be used to further our understanding of the biology of this devastating conditions.

### 3.3. Turner Syndrome and Klinefelter Syndrome

Mouse models with both paternal and maternally inherited 45,X karyotypes exhibit behavioural changes including reduced attention, growth retardation, and hearing defects, which resemble aspects of human Turner syndrome (TS) (reviewed by [33]). These models have been useful for understanding the X-parent-of-origin-effect on TS-associated phenotypes. However, 39,X mice do not manifest some TS-associated phenotypes, such as motor deficits; this may reflect differences in X-inactivation between mouse and humans. Mouse models of Klinefelter syndrome (XXY male) develop hypogonadism and cognitive problems and have impaired fertility (reviewed by [34]), phenotypes that resemble aspects of KS. Molecular studies undertaken in XXY mouse models have shed light on the possible chemical alterations in the brain that cause cognitive problems observed in KS [34], and this knowledge may lead to the development of therapeutic strategies.

## 4. Mouse Models of Segmental Aneuploidies 

Mouse models of segmental aneuploidy are invaluable for our understanding of which dosage-sensitive genes result in the deleterious phenotypes that are associated with these genomic changes in humans. Moreover, mouse models of segmental aneuploidy, not associated with a specific human syndrome, can also be used to understand the relationship between gene and phenotype. For example, mouse models with 0.8 Mb reciprocal chromosomal deletions and duplications have been used to identify the role of *Stat5* in immune-hypersensitivity and metabolic syndrome [35]. 

A number of models of Prader-Willi syndrome (PWS) (deletion of paternal 15q11–13) and Angelman syndrome (AS) (deletion of maternal 15q11–13) have been reported [36–38]. PWS is also associated with chromosome 15 maternal disomy and AS with paternal chromosome 15 disomy. Mouse models of these genetic changes have been reported and both exhibit reduced viability and neonatal growth retardation [36, 37]. Deficits in learning and memory have also been observed in a mouse model with a maternally inherited segmental deletions (*Ube3a-Gabrb3*) corresponding to part of the region lost in AS [38]. Mouse models deficient in the *Ube3a* and *Gabrb3* PWS/AS candidate genes exhibit neurodevelopment and behavior changes, highlighting the key role these genes play in the syndromes [39–41]. 

Interestingly, maternal duplications of the PWS/AS associated region, 15q11–13, are associated with autism [42]. A mouse model of the duplication of the mouse syntenic region of chromosome 7 exhibits some features that resemble autism, but only when the duplication is paternally inherited in contrast to the inheritance pattern observed in humans [43]. These models will help give insight into the genetic and biochemical abnormalities causing autism. 

Mouse models of DiGeorge syndrome (deletion of 1.5–3 Mb at 22q11) have been crucial to the molecular understanding of this condition. A series of complementary mouse models with full or partial deletions of the region syntenic with 22q11 identified the key deleted gene, *Tbx1*, responsible for the syndrome's deleterious phenotypes [44–46]. The 22q11 deletion is also the largest known genetic risk factors for schizophrenia [47, 48], and the DiGeorge mouse models may also be useful to further understanding of this condition [49]. Similarly, mouse models of the complete 3.7 Mb deletion and duplication associated with Smith-Magenis (SMS) and Potocki-Lupski (PTLS) syndromes have helped identify one of the key dosage sensitive genes, *Rai1* [50]. Moreover, these models have also been used to investigate the relative effect of genomic rearrangement versus gene copy number change on gene expression [51]. Work in this field has also highlighted the very complex interactions of genes both within the copy number altered region and those elsewhere in the genome [52], as the penetrance of some PTLS-like features in the mouse models vary with the size of the region disrupted and the genetic background of the model. The effect of genetic background on the penetrance of aneuploidy-associated phenotypes has also been highlighted in the Tc1 mouse model in which DS-like heart defects appear with a greater penetrance on a C57BL/6 mouse inbred line background [15]. 

A mouse model of Williams-Beuren syndrome (WBS) has been developed recently that exhibits a large number of informative neurodevelopmental and behavioural abnormalities [53]. This model is likely to be crucial to further understanding of WBS. 

## 5. Future of Aneuploid Mouse Models

Complete and partial mouse models of aneuploidy have significantly contributed to our understanding of the complex relationship between dosage of individual genes and the resulting phenotypes that arise in individual aneuploidy syndromes. Unfortunately, even for the most widely studied aneuploidy disorders such as DS, we are a long way from understanding much of the molecular basis of the pathology. However, the rate of progress in understanding the effects of gene copy number and expression levels is increasing, and we now know that there is a considerable variation of small genomic regions, copy number variation (CNV), across the entire human genome in normal individuals. These regions can be up to a megabase in size and affect much of normal human phenotypic variation, including susceptibility or resistance to common disorders (e.g., see [54–56]). New mouse models of CNVs will be beneficial to study not only the effects of gene dosage but also to dissect the effects of altering copy number for the regulatory elements found in these regions of the genome. 

Advances in our understanding of the human genome will present new opportunities for the development of novel mouse aneuploid models. Equally, findings from existing mouse models will continue to influence human genetic studies. Thus, complementary human and mouse genetic studies are key to unraveling the links between gene copy number and phenotype. 

## Figures and Tables

**Table 1:** Examples of mouse models of segmental aneuploidies.

Human syndrome	Associated genetic change	Aneuploid mouse models
Angelman syndrome	deletion of maternal 15q11–13	*PatDp* [37]
		*MatDf(Ube3a-Gabrb3)* [38]
Prader-Willi syndrome	deletion of paternal 15q11–13	*MatDp* [37]
Autism risk factor	Duplication 15q11–13	*matDp; pat Dp *[57]
Smith-Magenis syndrome	deletion of 17p11/17p11.2	*Df(11)17 *[58]
		*Df(11)17-1; Df(11)17-2; Df(11)17-3 * [59]
Potocki-Lupski syndrome	duplication of 17p11/17p11.2	*Dp(11)17* [58]
DiGeorge syndrome	deletion of 22q11.2	*Df1* [44]
		*Idd-Ctp *[45]
		*Idd-Arvcf *[46]
		*Df2; Df3; Df4; Df5 *[60]
Williams-Beuren	deletion of 7q11	*PD* and *DD* [53]
—	deletion/duplication of 17q21	*Df11[ 1] *and *Dp11[ 1]* [35]
